# Extracts and Gleanings

**Published:** 1875-02

**Authors:** 


### Extracts and Gleanings.

---

*Progress of Surgery.*—In a paper, read before the Missouri State Medical Society, by I. W. Trader, M.D., we find an interesting *resumé* of the Progress of Surgery in the last few years.

First is noticed the plan of Carl Esmarch, of Denmark, to insure bloodless operations; introduced into England by Mr. McCormick. It "consists in an elastic bandage some two inches wide and four or five yards long, that is applied to the limb, beginning at the toes or fingers and extending beyond the point of operation; sufficient tension is used to completely compress the blood vessels. A strong rubber band or tube is now firmly applied immediately above the bandage and securely fastened; the bandage is then removed, and the limb may now be operated upon without the loss of blood. Upon this plan many operations have been performed in the London hospitals—including excisions of the knee and elbow joints, amputations and the removal of dead bone; and in one case amputation was performed for gangrene of the foot, the precaution being taken of beginning the application of the elastic bandage several inches above the mortified part. No ill effects of any kind have hitherto been observed from the use of this contrivance. Although the duration of the operation has varied from a few minutes to a half an hour, and even more, during the whole of which time the circulation has been completely arrested, no evidence has been afforded of the formation of emboli or thrombi in any of the cases."

Dr. Eve, of Nashville, Tenn., it is stated, has used this method in four operations with complete success; also five successful cases of amputation in Bellevue Hospital had been reported.

He alludes to the fact that the *transfusion of blood* from one person to another is receiving a new impulse. Many successful operations have been made. In fifty-seven cases reported by Dr. Martin, forty-five recovered. The opinion is expressed that if a plan could be found in which the blood might be made to pass into the veins of the subject without in any way coming in contact with the external air, the success would be invariable.

Mention is made of the idea of substituting for the cold water irrigation, as a surgical dressing, that of warm water immersion, or warm water fomentations. "Nine cases, with favorable results, are reported, in all of which the warm bath or warm fomentations were used, and in no case did phlegmonous inflammation. gangrene, or pyemia ensue. In one case only did suppuration occur, and that was after the limb had been removed from the bath."

The method of removing tumors by an elastic ligature, introduced into England by Sir Henry Thompson, is detailed. It is said to be disapproved by Mr. Jno. Erichson, the great surgeon.

The idea of removing fluids and gases from the shut cavities by pneumatic aspiration is favorably mentioned. Even the thoracic cavities, in effusions, etc., into the pleura, the method is regarded safe.

"In the perfected apparatus of Dr. Dieulafoy we have a means of relieving these intrathoracic effusions by an operation simple and, at the same time, comparatively harmless. Not only the pleural cavity, but the pericardium, the bladder, the stomach, and the intestines, may be relieved in the same way. In strangulated hernia we may be enabled to reduce the tumor, after the ordinary means fail, by withdrawing the fluid from the sack by aspiration, or the accumulating gases from the bowel, relieving the tension and stricture caused thereby. The plan of the instrument is simple and easy of construction, yet I would not advise the improvising of a Davidson syringe, as some do, from the fact that the valves are always defective, and may allow the escape of fluid and air back as well as forth. The air pump of the common cupping apparatus will better answer the purpose, and the point of a hypodermic syringe, with a foot of small size elastic tubing, will enable any one to meet an emergency. The best valve is oil silk or thin rubber firmly bound to the valve seat by a small silk ligature."

R. C. W.

*Diseases of the Liver—Malarial Poisoning.*—By L. Washington, M.D., in *Pa. Jour. of Med.*—In the treatment of diseases of the liver, while the claims of podophyllin, leptandrin, and nitro-muriatic acid are not to be ignored, we should place Chionanthus Virginica, or old man's beard, first on our list of remedies. In all the various forms of liver complaint, incident to malarial poisoning, its action as a liver-regulator is undoubted. It is useful whenever digestion is enfeebled or nutrition is impaired, and whether the bowels is constipated or too loose. It seems to operate not alone as a stimulant to the liver, causing an increased flow of bile, nor yet alone is it a tonic, acting as an auxiliary to the functions of nutrition and assimilation, but it also exerts an alterative action upon the mucous membrane of the stomach and bowels, and upon the blood. In dropsical accumulations, jaundice, and malarial cachexia, it is the remedy *par excellence.* In that form of chronic malarial diseases known as obstinate and protracted intermittent fever, in which quinine has failed, you will find a certain cure and preventive of the return by giving fl. ext. chionanthus, a teaspoonful three or four times a day, alternating with—Cincho-quinine, ʒss.; prussiate of iron, ʒij.; sulph. strychnia, grs. ij. M. Ft. pil. No. 120. Sig. One pill three times a day. If there is induration of the spleen, give iodoform and phytolacca. Iodide of ammonium is also an excellent remedy for spleen enlargement, and does not cause the bad effects of iodide of potassium. By giving three-grain doses, twice a day, the enlargement of the gland speedily disappears. R. C. W.

*Milk as a Remedy.*—Considerable has been lately said in medical journals concerning the value of milk as a remedial agent in certain diseases. The *Milk Journal* states on the authority of Dr. Benjamin Clarke that, in the East Indies, warm milk is used to a great extent as a specific for diarrhœa. A pint every four hours will check the most violent diarrhœa, stomach-ache, incipient cholera, and dysentery. The milk should never be boiled, but only heated sufficiently to be agreeably warm, not too hot to drink. Milk which has been boiled is unfit for use. The writer gives several instances to show the value of this simple substance in arresting this disease, among which the following is to be noted: "It has never failed in curing in six or twelve hours, and I have tried it, I should think, fifty times. I have also given it to a dying man who had been subject to dysentery eight months, latterly accompanied by one continual diarrhœa, and it acted on him like a charm. In two days his diarrhœa was gone — in three weeks he became a hale, fat man. and now nothing that may hereafter occur will shake his faith in hot milk." A writer also communicates to the *Medical Times and Gazette* a statement of the value of milk in twenty-six cases of typhoid fever, in every one of which its great value was apparent. It checks diarrhœa, and cools the body. People suffering from disease require food quite as much as those in health. and much more so in certain diseases where there is rapid waste of the system. Frequently all ordinary food in certain diseases is rejected by the stomach, and even loathed by the patient; but Nature, ever beneficent, has furnished a food that in all diseases is beneficial — in some, directly curative. Such a food is milk. The writer in the journal last quoted, Dr. Alexander Yale, after giving particular observations upon the points above mentioned, viz., its action in checking diarrhœa, its nourishing properties, and its action in cooling the body, says: "We believe that milk nourishes in fever, promotes sleep, wards off delirium, soothes the intestines, and, in fine, is the *sine qua non* in typhoid fever." We have also lately tested the value of milk in scarlet fever, and learn that it is now recommended by the medical faculty in all cases of this often very distressing children's disease. Give all the milk the patient will take, even during the period of greatest fever; it keeps up the strength of the patient, acts well upon the stomach, and is every way a blessed thing in sickness.—*Boston Jour. Chemistry.*

*Atropia in Ophthalmic Affections.*—Mr. Hart, late Ophthalmic Surgeon to St. Mary's Hospital, says that we could, in the treatment of ophthalmic disease, better afford in this day—so far as our knowledge of disease and means of mastering it extend — to dispense with all other drugs, lotions and applications put together, than with atropia as a topical medicament. It allays local sensitiveness, and removes local spasm; it gives to the eye and to the internal muscular apparatus — iris and ciliary muscle — physiological rest, the greatest of all curative means. It is adapted to all diseased conditions of the eye, except oval dilatation of the iris in glaucoma.

The frequency of its use must vary with the facility and rapidity with which it acts. In the treatment of keratitis, and minor cases of deeper inflammation, one application a day, or at most two, will suffice, and presently once in two or three days. It will be enough then to keep the pupil dilated, and the ciliary apparatus at rest and free from tormenting spasm. His favorite "drop" or formula in the use of atropia is as follows: ℞. Neutral sulph. of atropia, grs. ii.; glycerin, gtt. v.; distil. water, ℥i. M.—*British Medical Journal.*

*Paralysis Caused by Worms.*—A little girl, aged five years, was attacked on the night of the 14th instant with apparent paralysis of motion, which had continued forty-eight hours, the patient being utterly unable to move hand or foot. There were no cerebral symptoms, and no fever. A swollen upper-lip, a dry cough, with a tendency to vomit, and a foul tongue, indicated the existence of worms, and the following prescription was made: ℞. Calomel, grs. viij.; santomine, gr. i.; fluid ext. pink-root, ʒss. M. and give immediately in a little syrup. The above dose was given at bed-time. It operated next morning, bringing away a large number of lumbricoids, and a number of thread-worms, with prompt and decided relief to the patient. R. C. W.

*Chloroform as a Liniment.*—As an anodyne liniment and revulsive, we have found nothing so prompt, convenient and efficient as chloroform, particularly in headache and neuralgia. Apply upon a folded handkerchief, firmly pressed to the part. The burning sensation is severe, but will cease on moving the handkerchief to another place. If retained too long at one point, it will blister the skin. This application as a revulsive, in conjunction with the following prescription internally, will relieve most cases of neuralgia: ℞. Bromide potassium, grs. xv.; deodorized tinct. opii, gtt. xv. M. S. Take every hour until relief follows. R. C. W.

*Bromide of Calcium.*—Dr. W. A. Hammond, in New York *Medical Journal,* regards the bromide of calcium as superior to either the bromides of sodium or potassium, in its hypnotic properties. He says: "In those exhausted conditions of the nervous system attended with great irritability, such as are frequently met with in hysterical women, and which are indicated by headache, vertigo, insomnia, and a mental condition of extreme excitement, bromide of calcium has proved, in my hands, of decided service." He recommends the following formula as excellent: ℞. Bromide of calcium, ℥i.; syr. lacto-phos. lime, ℥iv. M. ft. sol. Dose, a teaspoonful three times a day in a little water. R. C. W.

*Byrd on Cholera Infantum.*—Dr. Harvey L. Byrd writes as follows in the Philadelphia *Medical Times:* This terrible scourge of infancy and childhood is carrying large numbers of the young and tender ones of this community to their long homes, and such is the extent of its ravages that it might be said with propriety of language to prevail at this time as an epidemic in our midst.

Baltimore, hygienically considered, is probably equal in all, or at least very many respects, to her most favored sister cities; but, while this is the case, the hand of the destroyer occasionally falls heavily upon her, and she is then called upon to mourn the loss of those she cannot rescue from the embrace of death. Since the advent of Summer, the mortality has been considerable among infants and children one to three and four years old, but it is chiefly within the last three weeks that our mortuary tables exhibit a fearfully large proportion of death from cholera infantum. Within this period, there has been not only a steady but an alarming increase in the death statistics from this generally intractable and fatal malady. After resorting to the remedies most in vogue in the treatment of Summer-complaint, such as calomel in minute and moderately large doses, alone and in combination with Dover's powder, chalk, charcoal, etc., bismuth, magnesia, pepsin, tannic and gallic acids, acetate of lead, alum, nitrate of silver, creosote, pyroligneous acid, laudanum, etc., alone and in various combinations and mixtures, with indifferent and unsatisfactory results, even when strict attention was given to diet, fresh air, bathing, stimulants when called for, etc., it was finally decided to adopt a plan of treatment with special reference to an alterative action on the blood; at the same time giving strict attention to the skin with a view to the elimination of the poison, as far as might be, by this organ.

Accordingly, with the leading object in view, namely, an appeal to the blood primarily, sulphite of sodium and aromatic sulphuric acid were prescribed internally, and tepid or cold alkaline baths, according to indications, ordered externally; to which whisky or brandy was added when required.

One grain of the sulphite, with four drops of paregoric, was given in gum-water every two hours, to a child one year old, and the dose doubled for a child two years old, increasing or lessening it according to age and the anodyne effects of the paregoric, thus: ℞. Sulphite of sodium, grs. xvj.; powdered acacias, grs. xij.; camphorated tincture of opium, fl ʒj.; water, ℥ ij. M. Sig. One teaspoonful every two hours, to a child one year old, shaking the phial before using. One drop of elixir of vitriol in three spoonfuls of iced water was given, three times a day, to a child one year old; and the dose was increased one drop for each year, and lessened to one fourth or one-half drop when below one year of age. A tepid or cold bath, rendered alkaline with an ounce or more of carbonate of sodium, or potassium, or common salt, was used morning and night. In addition to the foregoing remedies, aromatic cataplasms were ordered, and kept applied over the entire stomach and abdomen. Cow's milk and farinaceous articles of food were not allowed, and scraped or finely-chopped beef, or lamb, raw or but partially cooked, or essence of beef (to which a small portion of brandy was added when required by the feebleness or prostration of the patient), was used as much as practicable as nourishment. Wine whey was allowed freely in the second stage of the disease, when it agreed with the patient. Infants were allowed the mother's milk, or that of a healthy wet-nurse, and fifteen to twenty drops of lime-water three or four times a day when the milk disag:eed. This plan of treatment has been pursued for the past two weeks, with complete success. In a small proportion of cases, quinine, in appropriate doses, was also administered when a tendency to periodicity was observed.—*London Medical Record.*

*The Suture of Tendons.*—Dr. Rochelt gives an account of some experiments performed by him with the view of determining the value of this operation. He performed his experiments upon the tendo Achillis of rabbits. The carbolized cat-cut suture was used. The results were very favorable; the use of the extremities being in all cases perfectly restored.—*Wiener Medical Presse.*

*Therapeutical Applications of Eucalyptus Globulus.*—At the last meeting of the San Francisco Medical Society, Dr. Pigné-Depuytren said he had used the eucalyptus in the French Hospital for a year, during which time many interesting results had been noted. In March last, a hundred of the small trees had been planted on the Hospital grounds. They had now reached the height of seven feet. He related the following cases:

A man had arteritis of the leg, succeeded by gangrene, which extended so high up as to render amputation impossible. In two weeks a large ulcer resulted, whose odor was horribly fetid. Everything in turn was employed to destroy this odor, but to no effect. At last a decoction of eucalyptus was resorted to, and, without exaggeration, in five minutes all fetor had disappeared. The decoction continued to be used with the same effect until death occurred, two or three weeks subsequently.

Another man, who had been under treatment in the Hospital for two months, with extensive, deep ulcer from varix, of a year's duration, had the decoction applied to the ulcer three times a day, with remarkable effect. In five or six days the ulcer was entirely covered with healthy granulations, and in a month it was entirely well.

A woman had been troubled for many months with an ulcer around the orifice of the urethra. It was cauterized five times with no result. After twelve days' use of the decoction of eucalyptus, washing thrice daily, it was well.

Four cases of syphilitic chancres healed under the eucalyptus dressing, without other treatment. These were very recent cases, or constitutional treatment would have been resorted to.

He had but one case of intermittent fever to report. This had proved rebellious to quinia, and also to arsenic, which latter had been administered for two weeks. A three weeks course of the eucalyptus cured entirely.

So numerous were the cases of bronchitis cured with the drug, that it was hardly worth while to mention them. A syrup made by adding sugar to the decoction, and evaporating, was here preferred.

The decoction was made by boiling twelve large leaves (green)

in a pint of water down to one third. The tincture was prepared from the dry leaves.

Dr. Pigné-Dupuytren added that he now always prescribed quinia in the infusion of eucalyptus, and considered such combination of great efficacy.

*Neuralgia.*—Dr. J. H. Johnson, of Providence, R. I., says: I am daily strengthened in the conviction that this disease is asthenic in character, although the sufferer may not necessarily be of a cachectic diathesis. Decay and partial loss of the teeth, with consequent difficulty in mastication, and, as a result, imperfect digestion, are among the chief causes which produce a lack of vital force, diminishing nerve nutrition and inducing the condition known to the profession as a neuralgic diathesis. In practice I daily meet with persons suffering from neuralgia, who, upon coming under prompt and vigorous tonic treatment, are speedily restored to a normal condition, the pains vanishing as the digestive organs resume tone and strength. For several years I have used the elixir phos. ferri, quin., et strych., which has never failed to produce the desired tonic result.

I now rely upon the croton chloral to give the patient relief from all neuralgic pains until the general system can be recuperated, and brought into a condition to defy this wearing, exhausting disease. The physician, in dealing with it, varies his treatment to meet the requirements of different constitutions.—*Boston Jour. of Chemistry.*

*Tuberculosis not Incurable.*—In a late communication to the Academy of Sciences of Paris, M. Metzquer tried to upset Villemin's doctrine. For the last five years the author has made experiments (from seventy to eighty), under the direction of Professor Feltz, of the Faculty of Nancy. He never succeeded in inducing pulmonary consumption in the inoculated animals. The results were capillary embolism, infarctus, vesicular pneumonia, etc., all of which lesions have (the author maintains) been confounded with tubercle. Tuberculosis may, however, be generated in animals (says M. Metzquer), without inoculation of tubercular matter, by rough treatment, bad blood, and, strange to say, by inflicting a wound upon them.—*Lancet.*

*Induction of Premature Labors.*—Dr. Jas. R. Swayne (*British Medical Journal*) reports twenty cases of induced premature labor. The instruments used were sponge tents and Barnes' dilators. The time occupied by the artificially induced labor varied from six hours to sixteen days. Out of twenty cases three mothers died. Two of these were in so desperate a condition that, without the operation, speedy death was inevitable. In only one could death be clearly the result of the operation. Eleven out of twenty infants were still-born. Of those born alive, two died. This great mortality is due to the frequent malposition of the child. On the whole, the Doctor's experience does not prove the operation more favorably to the mothers than the only other alternative, craniotomy. The saving of infant life is a great gain. Still, the responsibility is so great that it should be shared with another practitioner.—*Medical Examiner.*

*Galvano-Cautery.*—Perhaps the most striking results reported during the year in this department are those of Bryant in the use of the galvanic cautery in lupus, epithelioma and nævus. Four cases of lupus are given where a single thorough application of this means resulted in complete cure. Six cases of epithelial cancer were reported, in four of which but one operation was required, followed by rapid recovery. In one instance there was some recurrence at the end of nine months, which was removed in the same way; and in the other case, two minor cauterizations were required subsequently. The patients were under ether, and the porcelain cautery was generally used, or such a one as the case required.—*Archives of Dermatology.*

*Nelaton's Method of Resuscitation from Chloroform Narcosis.*—Dr. I. Marion Sims (*British Medical Journal*) most impressively details his observations respecting the practical workings of the above method. Briefly stated, it consists of reversing the body of the patient, either by hanging him up by the feet, or laying him over a bed or table, so that the blood from the greater part of the body can gravitate towards the head. The tongue is drawn forward by a tenaculum and artificial respiration tried. This practice is based on the theory that death from chloroform takes place from cerebral anæmia.—*Detroit Review of Medicine and Pharmacy.*

*The Curability of Syphilis.*—It is my firm conviction—indeed, I never doubted the fact—that syphilis is absolutely curable; that it is not a life-long disease, as some have maintained. It is generally admitted that, so long as a person is suffering from secondary syphilis, he is incapable of contracting an indurated sore, or any further constitutional infection. If, then, cases are met with in which, after a lapse of time, patients do contract a second indurated sore, followed in due course by a secondary general infection, they afford strong proof that the first attack has been radically cured. This is the view now entertained by Ricord, whose long experience has shown him many such cases. It is corroborated by the cases recently brought before the Royal Medical and Chirurgical Society by my colleague, Mr. Gascoyen, and I have myself seen repeated instances of the same thing. The curability of syphilis is also further evidenced by the number of persons, young men especially, who have suffered from it, and who afterwards marry and have perfectly healthy families.—LANE, *in British Med. Journal.*

*The Use of Koumis.*—It is stated in foreign journals that koumis has been employed in all the principal hospitals of Paris in affections of the respiratory organs, from mere catarrh to the third stage of phthisis, and that with excellent results. In affections of the digestive organs in persons suffering from albuminuria, it has also been very serviceable. In cases in which the disease is accompanied by diarrhœa, the koumis is given in a state of more advanced fermentation, koumis No. 2. The patients soon get accustomed to this drink, and afterwards take it with pleasure. Commencing at first with two or three glasses daily, the dose is gradually increased until two or three bottles or more are taken.—*Medical and Surgical Reporter.*

*New Application of the Marsh Mallow.*—According to a statement in *The Garden*, gypsum, mixed with four per cent. of powdered marsh mallow root, will harden in about one hour, and can then be sawed or turned and made into dominoes, dice, etc. With eight per cent. of marsh mallow, the hardness of the mass is increased, and allows of it being rolled out into thin plates, and painted or polished.—*Pharm. Journal.*

*The Obstetric Swing.*—The best contrivance is one which I shall venture to call my own, though it may, very likely, have been suggested before. It acts on the principle of directing muscular action, and consists of a sheet twisted loosely in the form of a rope, and tied together at the ends. Put the feet into the loop at the lower end, and push ; grasp the other end with the hand, and pull; the power exerted in this way is indefinite; it is the gymnastic paradox of trying to lift oneself, and may be practiced till the sheet or back gives way. Its effect in labor is surprising, and is immensely appreciated by patients. It brings the body and muscles into play. It relieves that distressing sense of helplessness which all women feel, by enabling them to help themselves. It shortens labor. It saves the use of instruments. Allow me to recommend it to your readers.—*Boston Med. and Surg. Journal.*

*Influence of Anæsthetics upon the Sexual Impressions of Females.* A writer in the *Lyon Medical Clinic* says it is a well-established fact that, occasionally, under the influence of ether or chloroform, an excitation of the sexual organs is produced, and a feeling is excited in the mind by this sensation which may make a woman believe that she has been subjected to violence. In illustration of this statement, the writer says that, during delivery, he placed the woman under chloroform. The sexual sensations of the woman were so vivid, that she accused him of having violated her. Yet her husband and a dozen women had been present the entire time of the delivery. Other illustrations are given, from which the wise moral is deduced, that "physicians should never administer ether or chloroform except in the presence of witnesses."—*Detroit Rev.*

*Diplopia and Ptosis with Syphilitic Paralysis and Muscular Atrophy.*—Anstie reports a case of a man, thirty-two years old, the subject of syphilitic disease, who one morning noticed a sudden diplopia and complete ptosis of the left upper lid, which improved under the iodide of potassium. In the following February, he was attacked by sudden pain and extremely rapid loss of power and substance in the right arm. By June, the triceps and flexors were atrophied to a few threads. By the use of iodide of potassium and faradization, the muscles regained their size and strengih in four weeks.—*Archives of Dermatology.*

*Croton Chloral.*—Dr. Oscar Liebreich, in the *British Medical Journal*, makes some important observations on the use of this new anæsthetic. He says it differs from chloral widely in some of its effects. A drachm of croton chloral, dissolved in water and swallowed, produces in fifteen to twenty minutes a deep sleep, with anæsthesia of the head. He has experimented on maniacs during an attack of mania. They remained sitting on their chairs in a deep sleep for two hours together. If anæsthesia had reached so high a degree through the use of hydrate of chloral, the patients would have dropped from their chairs, and their pulses and respirations would have been considerably retarded. In some cases of tic douleureux, the remarkable phenomenon is exhibited of pain ceasing before sleep sets in. But the remedy only acts as a palliative in this dreadful disease. However, its action is to be preferred to that of morphia. He has never observed any unfavorable effects of croton chloral on the stomach or any other organ in frequent experiments. The indications for the use of this remedy are to be found: 1. In cases where hydrate of chloral is inapplicable on account of heart disease; 2. In cases of neuralgia in the region of the *nervous trigeminus;* 3. In cases where very large doses of chloral are necessary to produce sleep. He there recommends the addition of croton chloral to hydrate of chloral.—*Druggists' Circular and Chemical Gazette.*

Dr. P. R. Palm, of Lehigh county, Pa., reports a case of abortion caused by the use of asarum canadense. It appears that this agent is largely resorted to in that locality. "When I asked her who advised her to use it, she said the girls use it, and that one of her female companions had used it for the same purpose, and with the same results. * * * * I also ascertained that large quantities of the asarum canadense are sold to women, especially to girls."

Shamful! Shame the necessity, mother of such an invention:
Could the contrivance die with them, well that they never be mothers.

[*N. O. Med. and Surg. Journal.*

*The Truth Comes Out.*—A man advertises for a competent person to undertake the sale of a new medicine, and adds that "it will prove highly lucrative to the undertaker."

*History of Cholera Summed Up.*—Dr. Ely McClellan (*American Practitioner*) sums up the mass of evidence, collected by a large number of observers, in the following propositions:

1. Cholera is a contagious disease, resulting from an organic matter which, entering into the alimentary canal, acts upon and destroys the epithelium.

2. The respiratory and digestive organs are the avenues by which individual infection is accomplished.

3. The active agents in the distribution of the cholera poison are the dejections of persons who have become infected, or of those in whom the disease is fully developed. In these dejections there exists an organic matter which in a certain state is capable of reproducing the disease.

4. Cholera dejecta, coming in contact with and drying upon any object, such as articles of furniture, bedding or clothing, will retain indefinitely its power, and under favorable circumstances the process of decomposition will at once commence.

5. Through the atmosphere of infected localities, the disease is frequently communicated to individuals.

6. Water, contaminated by surface-washings, by drainage from neglected sewers, cesspools or privies, may and does become contaminated with the cholera poison.

7. Without a combination of all the well-known factors of cholera, the disease cannot originate *de novo.*—*Detroit Review.*

*Heat from the Moon.*—The Earl of Rosse, as the result of an extended course of experiments, conducted with a view to determine whether there was any perceptible heat radiated or reflected from the moon, advances the opinion that not only does the moon reflect heat to the earth, but that this globe receives from its satellite by radiation a certain amount of heat by which it (the moon) was warmed. He estimates the heat thus reflected and radiated as the eighty-thousandth that obtained from the sun.—*Druggists' Circular.*

*Mammary Abscess.*—Quinine in full doses soon as chill occurs. Cease nursing at once, and remove the milk by hand rubbing, covering the parts with warm lard, and rubbing from the base of the gland towards the nipple.—*Medical Record.*

*Cremation.*—The manner in which Sir Henry Thompson's famous proposal has been taken up in all civilized countries leaves little room to doubt that cremation, as a means of disposing of the dead, will soon supersede inhumation. The German Cremation Society in New York, numbering about four hundred and fifty members, have decided on erecting a suitable hall, with walls of iron, sixty by forty-four feet, containing a rotunda supported by eight pillars. In the center there will be erected an altar for religious ceremony, and upon a large stand in front of this will be placed the coffin. The ceremonies ended, the coffin will be gradually lowered, by means of screws, into a furnace, where it will be submitted to a hot-air blast of one thousand degrees Fahrenheit. It is calculated that complete cremation would take place in an hour and a half, after which the coffin would be again returned to the altar. The ashes would then be gathered and placed in urns provided by the relatives of the deceased. Connected with the furnace there will be an apparatus for condensing the gases and smoke.—*Lancet.*

*Breathing in Rarefied Air.*—M. Paul Bert describes in *L' Institut*, a series of experiments made on himself in a receptacle in which the diminution of pressure of air was carried to 25 centimetres—equivalent to an altitude of nearly 1¾ miles. He found he could avoid all the ill effects of this diminished pressure by breathing a mixture of 60 per cent. oxygen with atmospheric air. This discovery has already been made trial of by æronauts, who will, by its means, be able to attain heights hitherto impossible.—*Baltimore Physician and Surgeon.*

*Camphor Water.*—The simple camphor water, *aqua camphora*, of the shops is one of the best and cheapest washes for the teeth and mouth. Its detersive power is great, leaving the mouth and gums very clean and sweet. It is fatal to parasites, and therefore discouraging to tartar and decay. It has also a healing influence upon aphthous sores (canker) of the mouth and throat. It lessens sensibility of the teeth, and tends to resolve the small abscesses known as gum-boils. We recommend it as a standard article of the toilet.—*C., in Boston Journal of Chemistry.*

*Acute Bright's Disease.*—Dry cupping over the loins and digitalis. The digitalis increases the blood pressure, causing more water to pass through the kidneys so as to flood them. If symptoms of incipient uremia are present, or perhaps the patient has had one or more convulsions before admission, hydragogue cathartics, diuretics, and dry cupping to the loins, are the chief measures for the relief of the convulsions or symptoms of convulsions. Hot-air baths are occasionally employed. Acetate of potass., in thirty-grain doses, is a diuretic very commonly employed.

Hypodermic injeetion of morphine, as recommended by Prof. Loomis, of New York, has been resorted to in one case for the purpose of controlling such convulsions. In that single case the convulsions were completely controlled. Two cases of acute Bright's disease had been treated with ergot. The symptoms were at once relieved, and the treatment regarded with favor.—*N. O. Med. and Surg. Journal.*

*Treatment of Typhoid Fever.*—Dr. Compin, finding it difficult to reconcile patients and their friends with the practice of cold immersion in fever, had recourse to the pardonable artifice of adding a few drops of phenic acid to the water beforehand. This premised, he practiced aspersion over the entire surface with the utmost advantage, thus lowering the fever and inducing such relief that the sufferer himself was anxious for its repetition in a few hours. The patient and his friends ascribed the relief to the phenic acid, and the practitioner had the satisfaction of securing the great advantages of cold aspersion without running counter to their prejudices. *L' Union Medicale.*

*The Champion Chloroform-Tippler.*—Dr. W. W. Parker related, at last meeting of the Virginia State Medical Society, the case of a man named Johnson, a blacksmith, who became addicted to chloroform-tippling. How much in quantity he swallowed is not stated, but he bought and drank three thousand dollars' worth in three years! His mind then became affected and he imagined himself tricked. Meanwhile he fattened fifty pounds, and on ceasing the habit lived fifteen years in good health, and died from natural causes.—*American Journal of Dental Science.*

*To Keep Cider Sweet.*—Professor Horsford, of Cambridge, recommends the following plan for preserving cider sweet:

"When the cider in the barrel is undergoing a lively fermentation, add as much white sugar as will be equal to half or three quarters of a pound to each gallon of cider, and let the fermentation proceed until the liquid attains the right taste to suit; then add an eighth to a quarter of an ounce of sulphite (not sulphate) of lime to each gallon of cider in the cask: first mixing the powder in about a quart of the cider, and then pouring it back into the cask, and giving it a thorough shaking or rolling. After standing bunged up for a few days, for the matter added to become incorporated with the cider, it may be bottled or used from the cask."—*Baltimore Physician and Surgeon.*

*Easy Parturition.*—Dr. B. G. McPhail, in a letter to the *Virginia Medical Monthly*, relates the following facts: A pregnant squaw, carrying a heavy burden, walked eight miles over a rugged, rocky mountain. During the following night, after a few groans and sharp screams, she gave birth to a child. At sunrise the mother straddled a pony and rode twelve miles over a frozen trail with her babe, neither of them suffering any injury."

At a ration day on the San Carlos reservation, a little commotion was observed among the squaws, one of whom was seen to lie down on some hay. This event was soon explained by the screams of an infant. In less than half an hour the mother claimed a ration for the little stranger.—*Detroit Review.*

*Subcutaneous Injection of Carbolic Acid in Traumatic Erysipelas.* Dr. Hirschberg relates the case of a slaughterman who accidentally cut his upper arm, the wound being about five inches long, and penetrating the muscles. A good deal of venous hemorrhage followed. On the second day a bad attack of erysipelas of the arm came on. A two-per-cent. solution of carbolic acid was injected subcutaneously at the upper boundary of the erysipelas, and next day all traces of erysipelas had disappeared. The injections were repeated five times within the next three days, and the wound healed far more rapidly and with much less suppuration than could have been expected.—*Berlin Woch.*

*New Researches on Podophyllin.*—In a note on the results obtained in the clinique of M. Demarquay from the use of podophyllin, M. Gerard Marchant remarks that the recent memori of Dr. Paul on the treatment of habitual constipation by this remedy has anew called the attention of practitioners to its value. Podophyllin is the resin obtained from the root of the *podophyllum peltatum,* a wild plant of North America, which is sometimes called the Carolina Ipecacuanha. It belongs to the class Berberidaceæ. Numerous experiments uudertaken by M. Delpech demonstrate that podophyllin is composed of two resins, one of which is soluble in chloroform, ether and alcohol, while the other is insoluble. It may be administered in the form of a pill combined with ginger or extract of hyoscyamus. Of forty patients to whom it was administered, three only were refractory to its action. One of these cases suffered from cancer of the uterus, a second from fibrous tumor of the uterus, and a third from stricture of the rectum. The effects of the administration of podophyllin are felt in about twelve hours. Its action is not accompanied by pain like that of aloes, nor by nausea like that of jalap, but by tickling sensations, gurgling, etc. The motions are semi-fluid, usually moulded, normal in color, and contain a considerable quantity of bile. Podophyllin may be employed for a long time without producing any secretory disturbance of the digestive tube, providing it is not given in too large doses. In the cases under M. Demarquay, which were chiefly women, the dose was never more than a grain, and no ill effects ever followed; but the dose of half a grain is preferable. —*Bulletin Génèrale de Thèrapeutique.*

*Injections of Cod-Liver Oil for Ascarides.*—The *Journal des Conuaissances Medicales* publishes a communication from Dr. Szerleki, of Mulhouse, on a case of irritation of the anus and adjoining parts, which was very greatly relieved by injecting an ounce of cod liver oil into the rectum.—*Obstetrical Journal.*

*To Promote Sound and Tranquil Sleep.*—Dr. Rittman recommends rubbing the abdomen with the hand for eight to fifteen minutes. A glass of cold water before retiring is also recommended for the same purpose.—*Baltimore Physician and Surgeon.*

*Muriate of Ammonia in Bronchitis, Catarrhal Pneumonia, etc.*—In obstinate acute bronchitis after the first intense stage, in catarrhal pneumonia both of children and adults, in bronchorrhœa, and also in ordinary chronic bronchitis, I have obtained more apparent good from the use of muriate of ammonia than any other remedy. Of course other secondary measures are to be vigorously used, counter-irritants, poultices, support or diminution of food supply, etc., as the case may call for. The best formula for giving the muriate, with which I am acquainted, is as follows: Take of ammonia muriat. ʒij.; extr. glycyrrhiz., ʒj.; mucil. acaciæ, aqua, aa f℥iij. M. S. Tablespoonful for an adultevery two hours; teaspoonful for a child a year old every three hours.

Sometimes, however, the patients object to the mixture of sweet and salt, preferring the following: Take of ammonia muriat., ʒij.; aqua, f℥vi. Dose as above.

Where the cough is very annoying, $\frac{1}{20}$th of a grain of sulphate of morphia, or 10 to 15 minims of tincture of hyoscyamus, may be added to each dose.

In bronchorrhœa the following may at the same time be used by inhalation twice or thrice daily: Take of sat. solution of alum, ℥vj.; tr. hyoscyam., ℥ss.—*New Remedies.*

*Lead in Soda Water.*—Our analysts are recommended by their brethren in Scotland to look after soda-water. Dr. Wallace, of Glasgow, found lead in the proportion of 9-100ths of a grain per gallon in a bottle submitted to him for analysis, which probably caused symptoms of lead poisoning in a lady who had been advised to drink soda-water freely, and who had indulged in this presumably harmless fluid to the extent of six or seven bottles per day.—*Baltimore Physician and Surgeon.*

*Tonic Prescription.*—The following prescription is regarded specially beneficial as a general tonic in syphilitic patients, and has been found to act exceedingly well in those cases in which the functions of the stomach have been almost destroyed by the use of alcoholics: ℞. Quinia sulph., gr. xxx.; tr. ferri chlorid., ʒiii.; strychnia sulph., gr. $\frac{1}{4}$; syr. zingiberis et aquæ ad., ℥iii. Teaspoonful every four hours.—*Medical Record.*

*Chronic Poisoning with Chloral Hydrate.*—Dr. Ludwig Kern, detailing a series of morbid phenomena which appeared in the course of a continuous employment of chloral, says: The most prominent symptoms were extensive cutaneous erythemas and pustular or papular exanthemata. The vessels of the face and head were first dilated; but in pronounced cases this condition extended downwards, following by preference the course of the nerve-trunks. Conjunctivitis, inflammations of the mucous membranes, and congestions in various parts of the body, were also noticed. A very important symptom was an interference with respiration, sometimes slight and sometimes amounting to actual dyspnœa, endangering life. The effect of chloral upon the skin and mucous membranes, and the dyspnœa produced by it, may all be explained by the assumption that it operates upon the vaso-motor center and the medulla oblongata, and that its paralyzing influence extends thence to the peripheral branches of the affected nerves.

There was another class of cases in which both the quality of the symptoms and their greater or less extension in the organism indicated a distinct change in the composition of the blood. They were attended with petechiæ, subcutaneous ecchymoses, colliquative diarrhœa, bronchitis, diffuse abscesses, high and continued fever, and all the symptoms of profound blood-poisoning.—*Practitioner.*

*Gonorrhœa—Local Remedies.*—Gonorrhœa may be treated successfully by local remedies only, and more successfully than by medicines taken internally; the injection must be weak enough and often enough used. A grain of sulphate of zinc to an ounce of water, used six or eight times a day, may be commenced with, and increased to two grains if necessary. If necessary, sulph. of copper may be tried instead, in the same strength.—*St. Louis Medical and Surgical Journal.*

*Quinine and Morphine.*—It had been noticed in several cases that, when one-quarter of a grain of morphine would not produce sleep, if ten grains of quinine were administered a short time previous to administering the morphine, the morphine would almost invariably act efficiently. This fact was noticed in connection with puerperal mania.—*Medical Record.*

*Arsenic in Malignant Lympho-Sarcoma.*—In four cases of malignant tumor treated with arsenic by Professor Czerney, the following results were obtained: The first case was an infiltrated epithelial carcinoma, occupying the whole buccal region of one side of the face; it had already caused swelling of the lymphatic glands. The three remaining were cases of malignant lymphoma.

In all of the cases, the forms in which the remedy was used was Fowler's Solution, five to thirty drops per day being given.

In the first case the internal administration of the remedy was alternated by parenchymatous injections. Occasionally the reaction was quite severe, and several abscesses formed. This patient completely recovered within seven months, and after the lapse of two years, no symptoms of the affection had returned.

In the second case, which commenced with enlargement of the tonsils and lymphatics of the neck, followed by swelling of the axillary, elbow, and femoral glands, the same treatment was pursued with like success. Dependence was principally placed, in this case, on the internal administration of the remedy, the dose being gradually increased until thirty drops per day were taken.

The third patient died of a scorbutic affection two months after beginning the treatment. The effect of the arsenic upon the swellings was manifest in their reduction. Death intervened in the fourth case before the effect of the arsenic was perceptible.—*Wien. Med. Wochensch.*

*Cardiac Œdema.*—In a lecture at La Charite, on diseases of the heart, Professor Sèe advocated the following treatment of œdema and anasarca due to heart affections: Extract of squills, fifteen grains; powder of squills, ten grains; for ten pills. From six to ten of these pills to be taken daily. At the same time about one drachm of bromide of potassium is administered daily. Under the influence of these two drugs the dropsy is stated to diminish rapidly.—*Druggists' Circular.*

*A Good Cement.*—A good cement for attaching paper labels to metal surfaces is composed of ten parts tragacanth mucilage, ten parts honey, and one part flour.—*St. Louis Medical and Surgical Journal.*

*Cholera.*—Dr. N. S. Davis places much reliance on the following mixture: ℞. Acid nitric, ʒi.; strychnia, gr. i.; tinct. opii, ʒiv.; syr. simplex, ℥i.; aqua, ℥iij. M. ʒi. every hour, alternating with calomel and morphine powders after every act of vomiting.

The above mixture is also valuable as a tonic in the convalescence from cholera, when there is nervous prostration and a general relaxed state of the system.

In the painless diarrhœas which so frequently precede the active onset of the cholera attack, and which seem to result from a generally relaxed condition of the mucous membrane, the following is a favorite mixture with some: ℞. Arom. sulph. acid, ʒiij.; sulphate magnesia, ʒiij.; tinct. opii, ʒiij.; aqua mentha, ℥iij. ʒi. dose, repeated more or less frequently, according to urgency of symptoms. It has the merit of being rather agreeable to the taste, and not likely to offend the stomach.

*Mucous Polypus cured by Acetic Acid.*—Dr. Méplain, of Moulins, gives the history of a man, thirty years of age, who had been afflicted for a month with mucous polypus of the velum palati. The tumor was growing rapidly, and impeded deglutition and speech, and gave rise to considerable hemorrhage. Applications of chromic and carbolic acids, removals with scissors, etc., afforded no relief, the tumor being constantly reproduced. A drop of acetic acid was injected into the polypus, and it immediately began to shrink; a second injection completed the cure, and five months later there were no symptoms of a return of the disease.—*New York Medical Journal.*

*Cod-Liver Oil Jelly.*—At a meeting of the Medical Society of Stockholm, Sweden, this article was exhibited as prepared by E. Queru, of New York city, and the following was stated to be its composition: Cod-liver oil, 85 parts; isinglass, 3 parts; sugar, 8 parts; water, 4 parts. As there exhibited, it was a semi-transparent jelly, of a yellowish-green color, having a strong odor, but less strong taste of the oil. The advantages claimed are: easy administration and complete assimilation, and its retention by the weakest stomach. A teaspoonful is said to equal a tablespoonful of the ordinary oil.

*Unstopping a Deaf Ear.*—Dr. C. S. Bacon (*Medical and Surgical Reporter*) says: C. G. B., aged sixty, complained of sudden deafness with the right ear, accompanied with a feeling of pressure on that side of the head, roaring and buzzing noise, etc. Examination of the meatus auditorius with speculum revealed a dark mass at or near the tympanum. Ordered injected into the meatus with a fountain syringe, small nozzle, nitrate potassæ, gr. x. in aqua, 98 Fah., one quart; in the evening to have dropped in the ear gtts. iv. of the following: ℞. Glycerin, ℥j.; acid carbolic, gtts. ij. M. And a pellet of wood to be kept in the ear. This treatment was repeated once each day to the 8th inst., at which time the injection brought away a large quantity of inspissated cerumen, with immediate relief of pressure, noises, and restoration of hearing.

*Camphor in Erysipelas.*—M. Revillout states (*Gaz. des Hopitaux,*) that he has several times had occasion to employ, with good result, in erysipelas an application used by M. Delpach at the Necker. It consists in painting the affected surface with a solution of camphor in ether (equal weights); and when this is employed in erysipelas of the face, and the affection has not yet reached the hairy scalp, its progress is usually arrested. It is also very useful in erythema caused by local irritation.—*Medical Times and Gazette.*

*Removal of Nitric Acid Stains.*—These well-known yellow stains can be removed either from the skin or from brown or black woolen garments, by moistening the spots for a while with permanganate of potash, and rinsing with water. A brownish stain of manganese remains, which may be removed from the skin by washing with aqueous solution of sulphurous acid. If the spots are old, they cannot be entirely removed.—*Druggists' Circular.*

*Treatment of Spasmodic Asthma.*—Dr. Julio J. Lamadrid recommends the combination of chloral hydrate with the bromide of potassium in the treatment of spasmodic asthma. The following is the formula which he employs: ℞. Chloral hydrat., ʒv.; potassi bromide, ʒijss.; syr. flor. aurontii, aqua dest., aa f. ℥i. M. Sig. A teaspoonful in half a wineglass of water every two hours, until sleep is induced or dyspnœa is relieved.—*Philadelphia Med. Times.*

*Treatment of Salivation by Atropia.*—The patient, a woman of sixty-eight years, had had two attacks of apoplexy, followed by hemiplegia of the left side. On being admitted into Dr. Ebstein's wards (Breslau Hospital), salivation was observed. According to the patient, it had begun a month previously. Atropia was administered internally without any effect. On the dose being increased, the quantity of saliva diminished. Atropia (the sulphate) was then injected hypodermically, and after seven minutes the salivation was stopped. On doubling the dose, the secretion was arrested for twelve hours. Dr. Ebstein explains the action of the drug through its influence on the permanent irritation of the secretory fibers of the salivary glands.

*Enemata of Bromide of Potassium in Obstinate Vomiting.*—Dr. Girabetti has obtained the very best results from the administration of enemata of bromide of potassium, in doses of from one-half to two drachms, in cases of obstinate vomiting attending the pregnant state. The same drug, also administered in enemata, has been very successful in the hands of Dr. Laborde, of Paris, in obstinate vomiting, connected with disease of the stomach, liver and intestines.—*Lancet.*

*Treatment of Pneumonia and Bronchitis by Carbolic Acid.*—Mr. Henry Greenway writes (*British Medical Journal*) that he has been treating uncomplicated pneumonia and bronchitis very successfully with carbolic acid. The following is his formula for an adult: ℞ Glycerini acidi carbolici ʒij.; extracti opii liquidi, *m*xxx.; aquæ camphoræ, ℥vj. M. S. A tablespoonful every four or six hours in water.

*Aconite in Headache.*—Dr. Fothergill (*British Medical Journal*) recommends the use of aconite in congestive headaches, attended with great cotemporary coldness of the hands and feet. The remedy dilates the peripheral blood-vessels, and so relieves the congested cerebral vessels.—*Nashville Jour. Med. and Surg.*

*Diabetes.*—Tannin is now recommended in diabetes. Minimum doses, ten grains, maximum from twenty to thirty grains, repeated two to four times per diem.—*The Archives.*

